# Investigating risk factors for methicillin-resistant *Staphylococcus aureus* and *Pseudomonas aeruginosa* in community acquired pneumonia: a model for using only electronic data capture

**DOI:** 10.1186/s41479-025-00188-6

**Published:** 2025-12-25

**Authors:** Philip Logan Whitfield, Kristen Wendler, Rachel Gabor, Mark Ridder

**Affiliations:** 1 Department of Clinical Pharmacy Services, Marshfield Clinic Health System and Marshfield Clinic Research Institute, 1000 North Oak Avenue, Marshfield, WI USA; 2 Biostatistics, Marshfield Clinic Health System and Marshfield Clinic Research Institute, 1000 North Oak Avenue, Marshfield, WI USA; 3 Infectious Diseases, Marshfield Clinic Health System and Marshfield Clinic Research Institute, 1000 North Oak Avenue, Marshfield, WI USA; 4 310 Maryknoll Avenue, Marshfield, WI USA

**Keywords:** Methicillin-resistant *Staphylococcus aureus*, MRSA, *Pseudomonas aeruginosa*, PSA, Community acquired pneumonia, CAP

## Abstract

**Background:**

The 2019 American Thoracic Society and Infectious Diseases Society of America community acquired pneumonia guidelines recommend empiric coverage of methicillin-resistant *Staphylococcus aureus* and *Pseudomonas aeruginosa* based on previous respiratory isolation, recent IV antibiotic use, and locally validated risk factors. This study aims to describe how local risk factors may be determined efficiently using data retrieved electronically.

**Methods:**

This retrospective cohort study focused on the time period May 13, 2020, through June 30, 2024. Consecutive adults admitted to one of five acute care facilities with confirmed community-acquired pneumonia were included. Community-acquired pneumonia was defined as the presence of one or more pneumonia diagnosis codes and an order for a respiratory culture or an antimicrobial with the indication of pneumonia or sepsis, 24 h before or within 48 h after the date and time of admission. Patients were excluded if they had a diagnosis code for hospital-acquired or ventilator-associated pneumonia, any subsequent admission in the study period, or if they had a previous respiratory culture positive for methicillin-resistant *Staphylococcus aureus* or *Pseudomonas aeruginosa* within a year of admission. The causative pathogen and the presence or absence of evaluated risk factors were electronically abstracted from billing data and health records. Serial quality assessments of electronic data were performed to improve accuracy until a well validated population was determined.

**Results:**

There were 4,558 unique patients included. Methicillin-resistant *Staphylococcus aureus* and *Pseudomonas aeruginosa* rates were 0.6% and 0.7%, respectively. Only age was inversely associated with risk of methicillin-resistant *Staphylococcus aureus* (OR = 0.86, 95% CI: 0.76–0.98). No significant risk factors for *Pseudomonas aeruginosa* were found.

**Conclusions:**

In rural or otherwise resource limited healthcare settings, risk factors for methicillin-resistant *Staphylococcus aureus* and *Pseudomonas aeruginosa* community-acquired pneumonia may be determined using only electronic data capture and the methodology described in this article.

**Supplementary Information:**

The online version contains supplementary material available at 10.1186/s41479-025-00188-6.

## Abstract (summary)

In rural or otherwise resource limited healthcare settings, risk factors for methicillin-resistant *Staphylococcus aureus* and *Pseudomonas aeruginosa* community-acquired pneumonia may be determined using only electronic data capture and the methodology described in this article.

**Supplementary Information:**

The online version contains supplementary material available at 10.1186/s41479-025-00188-6.

## Introduction

Community-acquired pneumonia (CAP) is the leading cause of infectious hospitalization and death in the United States [1]. Consequently, antimicrobial use for this infection exerts massive pressure on the metagenomic landscape of bacterial resistance across the U.S., and undoubtedly contributes to serious adverse effects and *Clostridioides difficile* infections. One mode of mitigating these consequences is selecting an empiric antimicrobial regimen that balances the effective treatment of the probable causative pathogens and avoids unnecessarily broad antimicrobial use. This task falls on the admitting provider when determining whether to utilize an antibiotic with activity against methicillin-resistant *Staphylococcus aureus* (MRSA) and/or *Pseudomonas aeruginosa* (PSA).

The 2019 Joint American Thoracic Society and Infectious Diseases Society of America (ATS/IDSA) CAP guidelines recommend coverage for MRSA and PSA if the patient has had a previous respiratory culture positive for these organisms, a recent hospitalization and IV antibiotic use within the past 90 days, or locally validated risk factors [2, 3]. This last, and most general recommendation, acknowledges that we live in a diverse landscape of healthcare, and apart from the former risk factors, existing data is contradictory on which patient characteristics are correlative with MRSA and PSA. To comply with the new recommendations for empiric coverage of MRSA and PSA, organizations need to invest the time and resources to identify their local risk factors. There have been a few studies published that have aimed to identify risk factors within a specific geographical region and/or for individual hospitals [4–8]. More research is needed to determine how specific local risk factors may be identified, especially within rural healthcare systems where resources are often limited, precluding manual chart review, and patient demographics may be very different from larger, urban, or academic health systems. The primary purpose of this study is to provide a roadmap to other institutions on how these risk factors for MRSA and PSA CAP may be manageably determined, ideally through electronic data capture alone. The primary endpoints of this study include determining the overall rate of MRSA and PSA CAP and identifying local risk factors for MRSA and PSA within a rural Wisconsin health system. Secondary endpoints include antimicrobials administered, all-cause mortality within 60 days of diagnosis, readmission within 30 days of discharge, and average length of stay.

## Methods

This retrospective cohort study focused on the time period May 13, 2020, through June 30, 2024. It was found to be exempt by the institution’s Institutional Review Board. During data retrieval and analysis ethical safeguards were ensured such as data anonymization and password protected data access.

### Patients

Included patients had to be 18 years or older, admitted with confirmed CAP in the study window, to one of five acute care facilities including our flagship referral hospital. Confirmed CAP was defined as the presence of one or more selected pneumonia diagnosis codes (Supplemental Table 1) in the electronic health record, and an order for a respiratory culture (e.g. sputum, endotracheal aspirate, bronchial alveolar lavage, etc.) or an antimicrobial with the indication of pneumonia or sepsis, 24 h before or within 48 h after the date and time of admission. Severe pneumonia was defined as direct admission to ICU or transfer to the ICU within 48 h. Antibiotic indications are a required field in the electronic health record, however only “pneumonia” exists without more specific identifiers of community-acquisition. Patients with a sub-diagnosis code of the ICD-10 codes listed in Table 1 of the supplement also were included (e.g., J18.9). A patient’s subsequent admission in the study period, patients with hospital-acquired (HAP) or ventilator-associated pneumonia (VAP) (ICD-10 codes of J95.851 and Y95) were excluded. Patients were also excluded if they had a previous respiratory culture positive for MRSA or PSA within the year prior to the index admission date.

**Table 1:** Baseline characteristics. Mean ± standard deviation reported for age and n (%) reported for categorical variables

	Frequency *N* = 4,558
**Age**	70.5 ± 15.8
**Sex**	
Female	2015 (44.2)
Male	2543 (55.8)
**Race**	
White	4421 (97.6)
Hispanic	69 (1.5)
American Indian or Alaska Native	41 (0.9)
Black or African American	15 (0.3)
Other^1^	55 (1.2)
**Severely Ill**	1064 (23.3)
**Site of Care (Staffed Beds)**	
1 Regional Referral (298)	2613 (57.3)
2 Regional (56)	868 (19)
3 Regional (40)	785 (17.2)
4 Regional (16)	151 (3.3)
5 Critical access (11)	141 (3.1)
**Causative organisms**	
Microbiology obtained	3946 (86.6)
Organism identified	1013 (22.2)
* Pseudomonas aeruginosa* (PSA)	30 (0.7)
Methicillin-resistant *Staphylococcus aureus* (MRSA)	27 (0.6)
No organism identified	3545 (77.8)
**Potential Risk Factors**	
Current or former tobacco/nicotine use	3143 (69.6)
Wound treatment	2399 (52.6)
Chronic kidney disease	1771 (38.9)
Diabetes (Type II)	1740 (38.2)
Chronic obstructive pulmonary disease	1615 (35.4)
ICU admission within 48 h	1064 (23.3)
Any COVID history	787 (17.3)
Alcohol abuse	750 (16.5)
Influenza (Prior to admission)	722 (15.8)
Concurrent COVID-19 diagnosis	707 (15.5)
Substance Use Disorders^2^	431 (9.5)
Immunodeficiency or HIV	395 (8.7)
Recent COVID-19 diagnosis	226 (5.0)
Bronchiectasis	188 (4.1)
End stage renal disease	182 (4.0)
Concurrent influenza	70 (1.5)
Pyothorax	38 (0.8)
Abscess of Lung/Mediastinum	30 (0.7)

^1^Includes Asian (24), biracial (23), and Native Hawaiian or other Pacific Islander (8)

^2^Includes opioids (207), non−psychoactive drugs (177), psychoactive drugs (146), stimulants (65), and cocaine (40)

### Other data acquisition and definition

Electronic data query of the EHR identified billing information and insurance claims, demographics, mortality, comorbidities (both chronic and acute), physician order details, culture results, antibiotics ordered, treatment duration, and length of stay. History of a comorbid condition was defined as presence of the specific ICD-10 within the EHR, billing information, or insurance claims any time prior to the start date of the index hospitalization. A concurrent influenza or SARS-CoV2 (COVID-19) infection was defined as the presence of the corresponding ICD-10 codes within the EHR, billing information, or insurance claims during the index hospitalization. Risk factors for examination originated from review of previous studies and guidelines, and from health system requests (Supplement Table 2). All microbiologic tests included for analysis and their frequency may be reviewed in Tables 3 and 4 of the supplement. These tests included respiratory cultures, blood cultures, urinary antigen tests, molecular respiratory panels, and other culture types potentially relevant in CAP (e.g. thoracentesis fluid). A concatenated list of all microbiologic results (both positive and negative) was reviewed and if necessary, assigned a discrete pathogen or pathogens. For example: a sputum culture result with freetext “Numerous *Pseudomonas aeruginosa* and Light Mixed Respiratory Flora” would have *Pseudomonas aeruginosa* attributed as the microbiologic result. A single patient could have multiple positive microbiologic results attributed (e.g. COVID-19 and MRSA).

**Table 2:** Risk of methicillin-resistant *Staphylococcus aureus* by multivariate regression analysis*

Risk factor	OR	95%CI	*p*-value
Concurrent COVID-19 diagnosis	0.46	0.10–2.01	0.327
Wound treatment	0.57	0.25–1.30	0.182
ICU admission within 48 h	0.58	0.20–1.71	0.327
Chronic obstructive pulmonary disease	0.76	0.31–1.87	0.556
Age (per 5 years)	**0.86**	**0.76–0.98**	**0.021**
Male	1.07	0.48–2.40	0.862
Any COVID-19 history	1.16	0.42–3.22	0.773
Immunodeficiency or HIV	1.18	0.35–4.06	0.787
Alcohol abuse	1.23	0.47–3.21	0.670
Diabetes (Type II)	1.43	0.61–3.36	0.412
Chronic kidney disease	1.55	0.63–3.83	0.342
Influenza (prior to admission)	1.83	0.74–4.54	0.194
Current or former tobacco/nicotine use	2.21	0.72–6.80	0.165
Substance use disorder^1^	2.38	0.90–6.25	0.080

^*^Risk factors that were infrequent (<5%) were not included in the model to prevent overfitting

^1^Includes opioids (207), non−psychoactive drugs (177), psychoactive drugs (146), stimulants (65), and cocaine (40)

**Table 3:** Risk of *Pseudomonas aeruginosa* by multivariate regression analysis*

Risk factor	OR	95%CI	*p*-value
Chronic kidney disease	0.37	0.13–1.05	0.062
Concurrent COVID-19 diagnosis	0.42	0.10–1.82	0.245
Immunodeficiency or HIV	0.49	0.07–3.65	0.485
Diabetes (Type II)	0.51	0.20–1.31	0.164
Wound treatment	0.58	0.26–1.25	0.164
Any COVID-19 history	0.82	0.24–2.82	0.756
Substance use disorder^1^	0.91	0.26–3.22	0.883
Age (per 5 years)	0.98	0.87–1.12	0.804
Alcohol abuse	0.98	0.40–2.43	0.967
ICU admission within 48 h	1.27	0.56–2.88	0.561
Current or former tobacco/nicotine use	1.63	0.58–4.55	0.356
Chronic obstructive pulmonary disease	1.94	0.87–4.32	0.105
Influenza (Prior to admission)	2.03	0.84–4.90	0.115
Male	2.21	0.96–5.07	0.061

^*^Risk factors that were infrequent (<5%) were not included in the model to prevent overfitting

^1^Includes opioids (207), non−psychoactive drugs (177), psychoactive drugs (146), stimulants (65), and cocaine (40)

**Table 4:** Antimicrobials administered

Antibiotic	*N* = 4,558 (%)
**Anti-Pseudomonal β-lactams**	**2544 (55.8)**
Cefepime	1322 (29.0)
Piperacillin-tazobactam	1143 (25.1)
Meropenem	69 (1.5)
Ceftazidime	10 (0.2)
**Anti-MRSA Agents**	**1076 (23.6)**
Vancomycin	1063 (23.3)
Linezolid	13 (0.3)
**Atypical Pathogen Agent**	**2924 (64.2)**
Azithromycin	2118 (46.5)
Doxycycline	806 (17.7)
**Non-Pseudomonal β-lactams**	**3562 (78.1)**
Ceftriaxone	2343 (51.4)
Ampicillin-sulbactam	333 (7.3)
Amoxicillin-clavulanate	592 (13.0)
Cefpodoxime	193 (4.2)
Cefuroxime	101 (2.2)
**Respiratory Fluoroquinolones**	**794 (17.4)**
Levofloxacin	793 (17.4)
Moxifloxacin	1 (0.0)
**Anaerobic Agents**	**398 (8.7)**
Metronidazole	372 (8.2)
Clindamycin	26 (0.6)
**Other Agents**	**262 (5.7)**

### Quality assurance

A quality assurance review was first carried out in summary and small batch format, focusing on correctable data quality issues. Serial review of distinct values and targeted samples continued with adjustment of programming until achieving satisfactory data quality. Subsequently, a random sample of 30 patients were manually reviewed in their entirety (mortality, baseline demographics of age at admission, sex, race, and ethnicity, tobacco use on admission, presence or absence of comorbidities, next admission date, culture results, and if confirmed definition of CAP was met) and compared to the data points abstracted electronically to verify accuracy. Assessment of these quality assurance endpoints include percentage of data agreements and total number of coding disagreements and their types. To maintain data integrity, data were not changed but rather designated as contributing or non-contributing to the final analysis for that measure.

### Statistics

Patient characteristics were reported using mean and standard deviation for continuous variables and counts and percentages for categorical variables. Characteristics of patients with CAP caused by MRSA were compared to patients with CAP caused by another pathogen using t-tests and Fisher’s exact test. Comparisons were also made between patients with CAP caused by PSA and patients with CAP caused by another pathogen. Multivariate logistic regression models, one for MRSA and one for PSA, were used to identify risk factors and estimate effect sizes after adjusting for other risk factors. To prevent overfitting, factors that occurred in less than 5% of the study population were not included in the models, and substance use disorder was condensed into a single factor. Model fit was assessed using the Hosmer-Lemeshow goodness of fit test and found to be acceptable. Multicollinearity was assessed using variance inflation factors (VIF), with all VIFS near one. Odds ratios, 95% confidence intervals, and p-values were reported alongside forest plots.

Differences in secondary outcomes, whether antibiotics were prescribed, days prescribed, length of stay, 30-day readmission, and 60-day readmission were reported using medians and interquartile ranges and counts and percentages. Comparisons between MRSA cases and other CAP cases and between PSA cases and other CAP cases were made using Wilcoxon rank-sum test and Fisher’s exact test, as appropriate.

Finally, counts and percentages of patients receiving specific antibiotics were reported. Differences between MRSA or PSA cases and other CAP cases were evaluated using Fisher’s exact test. All tests were two-tailed with *p* < 0.05 considered significant. Analyses were completed in R 4.1.1 with the following packages: lubridate, propCIs, tidywork, gt, and patchwork.

## Results

A total of 4,558 unique patients met inclusion criteria and were examined in the final analysis. The average age of the total population was 70.5 years, while the average age was significantly lower for those with MRSA CAP at 61.4 years (*p* = 0.015). In patients identified as having PSA CAP the average age was 68.3 years (*p* = 0.470). Overall, more patients were male (55.8%), a majority were white (97.6%), and almost a quarter had severe pneumonia (23.3%). Table 1 further summarizes the demographics of included patients.

**Fig. 1:**
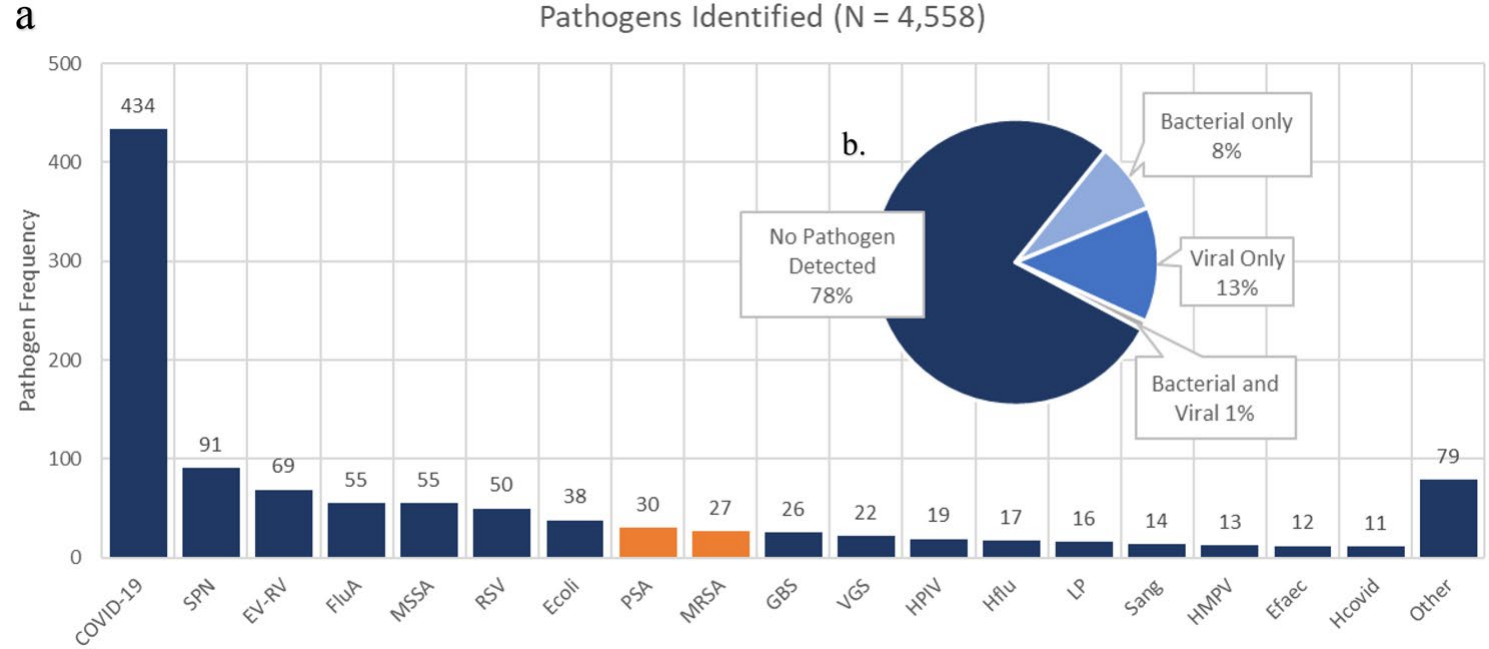
Alt text: (**a**) A pie chart depicting the incidence of no organism detection, viral, bacterial, and organism co-identification. (**b**) A bar graph depicting the incidence of viruses and bacteria identified as the cause of community-acquired pneumonia. Abbreviations: SPN; *Streptococcus pneumoniae*, EV-RV; enterovirus-rhinovirus, FluA; Influenza A, MSSA; methicillin-susceptible *Staphylococcus aureus*, RSV; Respiratory Syncytial Virus, Ecoli; *Escherichia coli*, PSA; *Pseudomonas aeruginosa*, MRSA; methicillin-resistant *Staphylococcus aureus*, GBS; Group B *Streptococci*, VGS; Viridans Group *Streptococci*, HPIV; Human Parainfluenza Virus, Hflu; *Haemophilus influenzae*, LP; *Legionella pneumophila*, Sang; *Streptococcus anginosus*, HMPV; Human Metapneumovirus, Efaec; *Enterococcus faecalis*, Hcovid; seasonal Coronavirus. Other organisms include Moraxella spp. (9), *Enterobacter cloacae complex* (8), *Klebsiella pneumoniae* (8), *Proteus spp*. (6), *Streptococcus pyogenes* (5), *Clostridium spp*. (4), *Fusobacterium spp*. (4), *Bacteroides spp*. (3), *Blastomyces spp*. (3), *Rothia mucilaginosa* (3), *Salmonella spp*. (3), *Klebsiella oxytoca* (3), *Adenovirus* (2), *Enterococcus faecium* (2), *Parvimonas spp*. (2), *Raoultella orthinolytica* (2), *Stenotrophomonas spp*. (2), Influenza B (1), *Cryptococcus spp*. (1), *Histoplasma spp*. (1), *Listeria monocytogenes* (1), *Morganella morganii* (1), *Mycobacterium avium complex* (1), *Pasturella multocida* (1), *Prevotella spp*. (1), *Pseudomonas stutzeri* (1), and *Serratia marcescens* (1)

A total of 3,946 patients, or 86.6% of the total population, had a microbiologic test obtained. Of those with microbiologic tests obtained, 1,013 or 22.2% of the total population had an organism identified. The most common organisms identified were SARS-CoV2 (9.5%), *Streptococcus pneumoniae* (2%), Enterovirus/Rhinovirus (1.5%), Influenza A (1.2%), and methicillin-susceptible *Staphylococcus aureus* (1.2%). There were 27 patients identified to have MRSA CAP (0.6%), and 30 were identified to have PSA CAP (0.7%). Further categorization of identified organisms may be found on Fig. 1.

### MRSA CAP

The results of the multivariate logistic regression analysis aimed to identify local risk factors for MRSA CAP are summarized in Table 2 and Supplemental Fig. 1 in graphical format. The univariate analysis may be found in Supplemental Table 5. Patient age was the only factor having a statistically significant inverse relationship with odds of MRSA CAP (OR = 0.86, 95% CI: 0.76–0.98). In the univariate analysis, end stage renal disease and history of a substance use disorder were associated with an increased odds of MRSA CAP, however this correlation was not found in the multivariate analysis when controlled for concurrent or COVID-19 history, wound treatment, chronic obstructive pulmonary disease, age, immunodeficiency or HIV, alcohol abuse, type 2 diabetes, chronic kidney disease, influenza prior to admission, and current or former nicotine dependence. Among MRSA cases, the median length of inpatient stay was significantly longer (non-MRSA: 5 days versus MRSA: 9 days, *p* = 0.005). The 60-day all-cause mortality rate was also significantly higher among MRSA patients (non-MRSA: 19.5% versus MRSA: 40.7%, *p* = 0.012). A full summary of secondary outcomes is summarized in Table 6 of the supplement.

### PSA CAP

The results of the multivariate logistic regression analysis aimed to identify local risk factors for PSA CAP are summarized in Table 3 and in Supplemental Fig. 2 in graphical format. The univariate analysis may be found in Table 7 of the supplement. In the univariate analysis, chronic kidney disease and Type 2 diabetes were negatively correlated with the odds of PSA pneumonia. These associations were not found in the multivariate analysis where no risk factors were associated with increased or decreased odds of PSA pneumonia. Among PSA cases, the 30-day readmission rate was significantly higher (PSA: 26.7% versus non-PSA: 12.9% days, *p* = 0.048). A full summary of secondary outcomes is summarized in Table 8 of the supplement.

### Antibiotics administered

The most common individual antimicrobials administered in the study population was ceftriaxone (51.4%) and azithromycin (46.5%). Anti-MRSA agents (vancomycin and linezolid) were administered in a combined 23.6% of patients while anti-PSA agents (cefepime, piperacillin-tazobactam, ceftazidime, and meropenem) were administered in a combined 55.8% of patients. An additional 17.4% of patients were administered a fluoroquinolone with anti-PSA activity. Complete records of administered antimicrobials may be found in Table 4.

### Quality analysis of data pull

In total, 34 electronically pulled data points were assessed for 30 patients. End-points assessed included mortality, age at admission, sex, race, ethnicity, tobacco use on admission, admission date, next admission date, if CAP definition was adequately assessed, 21 potential risk factors, length of stay, antibiotics ordered, duration of antibiotics, and culture results. A total of 13 coding disagreements and 1,007 agreements (98.7%) were identified. Three of the 13 disagreements existed because information within the EHR did not extend back far enough to when the diagnosis code for the comorbidity was first present within the patient’s health, billing, or insurance records; therefore, the diagnosis could not be confirmed. Most common coding disagreements occurred regarding tobacco use on admission (*N* = 3), history of substance use disorders (*N* = 2), history of a previous influenza infection (*N* = 2), and antibiotics ordered inpatient (*N* = 2).

## Discussion

This study was able to successfully determine the rates of and the local risk factors associated with MRSA and PSA CAP utilizing only electronic data capture. Institutions would need only to copy the methods of this study, tailoring data pull to their own context, to successfully report on similar results. The conclusions of our study; the rarity of MRSA (0.6%) and PSA (0.7%) as a cause of CAP, the discordance between these rates and the use of anti-MRSA (23.6%) and anti-PSA (55.8%) antimicrobials, and only age being inversely correlated with risk of MRSA CAP, may not be as interesting to the reader as how the data was obtained, and then how it was used to practically change patient care. The external validity of any identified local risk factor is poor by design, and any institution repeating this study should expect different results.

### Incidence and risk factors

The first conclusion of our data, the rarity of MRSA (0.6%) and PSA (0.7%) as a cause of CAP, will be used to calibrate providers’ expectations of how often they will encounter these pathogens. We believe the methods used to identify these pathogens are accurate for several reasons. First, these rates are in line with, though on the lower end, reported in the literature (MRSA 0.5% − 4.8%; PSA 0.35% − 4%) [7–12]. The lower incidence of MRSA CAP may be largely related to baseline MRSA rates in the community. The antibiogram in 2024 for our rural Wisconsin health system shows that 28% (*n* = 1,905) of all *Staphylococcus aureus* isolates were methicillin-resistant and this has been consistent year to year. Our MRSA rate is lower than the Wisconsin statewide average of 38% [13]. Interestingly, our rate of Staphylococcus aureus (both MRSA and MSSA) and PSA were strikingly similar to perhaps the most robust epidemiologic CAP study to date, the 2015 EPIC Study [14]. In that study *Staphylococcus aureus* was a cause of approximately 1.6% (versus 1.8% in this study) of patients and PSA was a cause of approximately 0.4% (versus 0.7% in this study).

Regarding the risk factors for MRSA and PSA CAP identified, only age was inversely correlated with incidence of MRSA while no factor was associated with PSA. Age as inversely proportional to risk of MRSA CAP does not easily translate into clinical guidance for providers. Furthermore, age has not been identified as a risk in other studies. We hypothesized that age is a proxy for substance use disorder, a risk factor that trended towards correlation with MRSA CAP, though did not reach statistical significance. On further analysis, we determined that if patients ≥ 72 years of age are excluded, a rerun of the multivariate model finds that substance use disorder suddenly has a statistically significant correlation with MRSA CAP (OR 3.01; 1.05–8.68) and age (every 5 years) does not (OR 0.98; 0.95–1.02). Further details are available in supplemental Table 9. This re-run excluded 7 patients aged 72 and older that had MRSA pneumonia, none of which also had a substance use disorder. Therefore, the authors agreed that even though this trend was not statistically significant in the original analysis, it is clinically meaningful.

All risk factors analyzed (supplemental Table 2) were selected because they have each been listed as criteria for healthcare-associated pneumonia, or HCAP, in previous editions of guidelines or investigated in other primary literature [3, 15–17]. We decided to exclude patients with a history of MRSA or PSA in a respiratory specimen and hospitalization with receipt of IV antibiotics in the last 90 days, as both are recommended in the most recent version of the ATS/IDSA CAP guidelines based on “consistent and strong” evidence for risk of these pathogens [18–20]. 

Despite selecting commonly discussed risk factors, our results differ from similar studies. One example, a retrospective cohort study by Lewis in 2021, examined the local risk factors for MRSA and PSA CAP with similar study design [4]. Lewis found illicit substance abuse, lung abscess/empyema, influenza, end stage renal disease, and chronic obstructive pulmonary disease (COPD) to be associated with MRSA. Lewis found that lung abscess/empyema, bronchiectasis, alcohol use disorder, and COPD were associated with PSA. Despite the variance in statistically significant risk factors, they occurred at very similar rates in the overall populations. Undoubtedly, the COVID-19 pandemic is a glaring difference between Lewis and this study. Considering the time frame of our study spans the worst of the COVID-19 pandemic, we were able to confidently evaluate COVID-19 as a risk factor, with any COVID-19 history (17.3%), recent COVID diagnosis (5.0%), and concurrent COVID-19 diagnosis (15.5%), being very common.

Our results further identified a stark discordance between these observed rates of MRSA and PSA CAP and the empiric use of anti-MRSA (23.6%) and anti-PSA antimicrobials (55.8%). We hypothesize that these high rates of antimicrobials targeting MRSA and PSA were attributable to providers’ perception that these pathogens are common, and a persistence in use of the HCAP designation is useful. A study out of the Department of Veterans Affairs in 2015 by Jones et al., hypothesized that the HCAP designation was a potential reason for significant increases in anti-MRSA and anti-PSA antimicrobial use [21]. After the publication of the 2005 ATS/IDSA HCAP guideline, they report a statistically significant increase in utilization of both vancomycin and piperacillin-tazobactam for the treatment of pneumonia from 2006 to 2010 (vancomycin: 16% to 31%; piperacillin-tazobactam: 16% to 27%) without a comparable incidence of MRSA and PSA as the cause. The percentage of positive MRSA and PSA cultures were found to be 2.2% and 2.0%, respectively. In a follow-up study to the aforementioned EPIC Study Team publication, rates of anti-MRSA and anti-PSA antimicrobials were lower at 17.7% and 12.3% respectively [22]. Importantly this study only included antimicrobials administered in the first 24 h of admission, excluded patients with recent hospitalization and nursing home residency, and had a strict definition of CAP requiring clinical signs and symptoms and chest radiography. Regardless of the difference, over a fifth of included patients receiving an anti-MRSA agent and over half receiving an anti-PSA agent places patients at serious risk of adverse events attributable to broad-spectrum antimicrobial use, opportunistic fungal infections, and development of multi-drug-resistant organisms such as VRE or ESBL producing *Enterobacterales*. As an antimicrobial stewardship effort, simply sharing preliminary data with the hospitalist groups across the included institutions has already significantly reduced use of anti-MRSA and anti-PSA agents for CAP.

### Methods of analysis

The utilization of exclusively electronic data capture through standardized coding systems ensured that the study population could be quickly identified over a larger study period in our resource limited setting. We reference “resource limited setting” very intentionally, as more robust methods of data analysis (i.e. manual chart abstraction) would require funding, staffing, and time not readily allocated to the antimicrobial stewardship programs of rural institutions already struggling to survive in the modern healthcare environment. For example, at our institution there is one full-time pharmacist as director of an antimicrobial stewardship program for 11 hospitals and 65 + ambulatory clinics and urgent care centers. There is no time allocated to physicians, microbiologists, infection preventionists, or data analysts. Rural or otherwise resource limited institutions could therefore benefit from the methods presented in this study. Despite the benefits to resource limited institutions, these electronic methods have inherent limitations. Firstly, the specificity of our definition “confirmed CAP” may be called into question as it relies on the presence of ICD-10 billing codes; the reader should be aware that a code does not exist specifically for *community-acquired* pneumonia. One recent study by Skull et al. out of Australia found that the positive-predictive value (PPV) of ICD-10 codes related to pneumonia was as high as 97.8% when compared to provider notation in the medical record [23]. However, when the ICD-10 code was compared to the chest radiograph report of pneumonia, it fell to 62%. We did attempt to increase the specificity for pneumonia by also requiring an order for a respiratory culture or an antimicrobial with the indication of pneumonia or sepsis, 24 h before or within 48 h after the date and time of admission. To date, the Lewis study has had the most comprehensive description of methods related to MRSA and PSA risk factor analysis. In that study, the investigator was able to use coded data via QualityAdvisor (Premier Incorporated, Charlotte, NC), a third-party product that overlays the electronic health record and “captures resource utilization, ICD-10 coding, and patient-related outcomes” [21]. In our own rural health system, and likely in others, clinical decision support software such as QualityAdvisor may not be available, and our unique methods of data abstraction would be useful. In any case it is advised that some method be used beyond reliance on an ICD-10 code, and we believe our approach was both precise and manageable.

Further limitations are the inability to fully evaluate competing diagnoses (e.g. pneumonia and urinary tract infection), to assure completeness of coding, and to attest accurate and timely charting. We believe that some of these concerns may be assuaged by the high data fidelity found in the manual quality assurance review. Respective to what may have been missed in manual review, insight was gained during widespread summary review. The study team would investigate possible errors when broken expectations in the data were found. One example of a broken expectation that was reviewed and solved is how approximately 98% of the total study population was electronically evaluated to have been prescribed an antibiotic for CAP, even though clinically all patients were expected to. Belonging to the 2% were patients prescribed an antibiotic for a concurrent infection that would also cover organisms likely to cause CAP, but where indication was not “pneumonia” or “sepsis” but the competing diagnosis. There were also competing circumstances like existing patient refusal/preference or end-of-life care, which correctly resulted in non-ordering of cultures or antimicrobials. Attention to confounding or competing factors in clinical decision-making and EHR data complexity should continue to be acknowledged in future research. Finally, a distinction is to be made between “anti-PSA” and more broadly coverage for multidrug-resistant Gram-negative rods as referenced in the ATS CAP guideline. There are some organisms that may, albeit rarely, cause CAP that are either intrinsically or likely resistant to ceftriaxone, but are not normally considered ESBL producing *Enterobacterales;* non-aeruginosa *Pseudomonas spp*., *Acinetobacter baumanii*,* Achromobacter xylosoxidans* to name a few. In our study we only had *P. aeruginosa* in view. If a facility is known to have a high relative amount of these organisms as a cause of CAP, they should consider enrolling them into a risk factor analysis of multidrug-resistant Gram-negative rods rather than P. aeruginosa alone. The ATS CAP guidelines recommend coverage for ESBL-producing *Enterobacterales* only on the basis of patient or local microbiologic (antibiogram) data and therefore are out of the scope of this type of analysis.

## Conclusion

This study found that age is inversely correlated with higher rates of MRSA CAP, and the absolute rate of MRSA and PSA were very low. Furthermore, the exceedingly high use of anti-MRSA and anti-PSA antimicrobials empirically for CAP identified an opportunity for antimicrobial stewardship intervention. Possibly more important to the reader, this study showed that a patient population of interest and corresponding demographics, co-morbid conditions, and information related to a clinical course can be quickly and accurately abstracted electronically to evaluate for MRSA and PSA CAP risk factors. However, electronic data abstraction comes with its limitations, including the inability to use clinical judgement to assess different endpoints, such as antibiotic orders and culture results, and the reliance on ICD-10 codes that may be too broad or not appropriate for a specific patient case. Future studies could aim to explore ways to more accurately capture patients with CAP electronically.

## Electronic supplementary material

Below is the link to the electronic supplementary material.

Supplementary Material 1

## Data Availability

The datasets used and/or analyzed during the current study are available from the corresponding author on reasonable request.

## Abbreviations

ATS/IDSA: American Thoracic Society and Infectious Diseases Society of America
CDC: Centers for Diseases Control and Prevention
CAP: Community–acquired pneumonia
EHR: Electronic Health Record
HCAP: Healthcare–associated pneumonia
HIV: Human Immunodeficiency Virus
HAP: Hospital–acquired pneumonia
ICD: 10–International classification of diseases
MRSA: Methicillin–resistant Staphylococcus aureus
PSA: Pseudomonas aeruginosa
VAP: Ventilator–associated pneumonia
