# Supplementary material for: Investigating risk factors for methicillin-resistant Staphylococcus aureus and Pseudomonas aeruginosa in community acquired pneumonia: a model for using only electronic data capture
Source: Pneumonia (Nathan). 2025 Dec 25;17:32. doi: 10.1186/s41479-025-00188-6 (PMC12739841; doi:10.1186/s41479-025-00188-6)
Supplement: Supplementary file 1 — Supplementary Material 1 [file 41479_2025_188_MOESM1_ESM.docx]

**Supplement**

| **Supplemental Table 1.** Pneumonia international statistical classification of diseases, tenth revision code (ICD-10). Sub-diagnosis codes of the codes listed above were also included (e.g. J18.9) | |
| --- | --- |
| **Type of Pneumonia** | **International Statistical Classification of Diseases, Tenth Revision Code** |
| Pneumonia caused by *Streptococcus pneumoniae* | J13 |
| Pneumonia caused by *Haemophilus influenzae* | J14 |
| Pneumonia, not elsewhere classified | J15 |
| Pneumonia, unspecified organism | J18 |
| Pneumonitis caused by solids and liquids | J69.0 |
| Legionnaires Disease | A48.1 |
| Pneumonia caused by infectious organisms, not elsewhere classified | J16 |
| Pneumonia in diseases classified elsewhere | J17 |

| **Supplemental Table 2.** Co-morbid conditions investigated as risk factors for methicillin-resistant *Staphylococcus aureus* (MRSA) and *Pseudomonas aeruginosa* (PSA) | |
| --- | --- |
| **Investigated Risk Factor** | ***International Statistical Classification of Diseases, Tenth Revision* code** |
| Human Immunodeficiency Virus (HIV) | B20 |
| Bronchiectasis | J47 |
| Chronic Kidney Disease | N18 |
| End-Stage Renal Disease | N18.6 |
| Influenza caused by certain identified influenza viruses | J09 |
| Influenza caused by other identified influenza virus | J10 |
| Influenza caused by unidentified influenza virus | J11 |
| Type 2 Diabetes Mellitus | E11 |
| Chronic Obstructive Pulmonary Disease | J44 |
| Tobacco Use | Z72.0 |
| Substance Use Disorder-Psychoactive | F19 |
| Substance Use Disorder-Opioid | F11 |
| Substance Use Disorder-Cocaine | F14 |
| Substance Use Disorder-Use of Non-Psychoactive Substance | F55 |
| Substance Use Disorder-Stimulants | F15 |
| Alcohol Related Disorders | F10 |
| Abscess of the lung and mediastinum | J85 |
| Pyothorax | J86 |
| Wound Treatment | L00-L08 |
| Immunodeficiency | D80-D89 |
| COVID-19 Infection | U07.1 |
| Pneumonia due to COVID-19 | J12.82 |
| Personal History of COVID-19, unspecified | U08.9 |
| Post COVID-19 Condition | U09.9 |

| **Supplemental Table 3.** Types of molecular and antigen tests included in the study. These fields are those present in the electronic health record | | |
| --- | --- | --- |
| **Molecular Targets (Nasopharyngeal Swab)** | | |
| Panel Name | Frequency Obtained  N = 4,558 | Targets |
| Abbreviated Respiratory Pathogen Panel | 85.8% | Influenza A |
|  |  | Influenza B |
|  |  | Respiratory Syncytial Virus |
|  |  | SARS-CoV2 (COVID-19) |
| Full Respiratory Pathogen Panel | 23.8% | Adenovirus |
|  |  | Enterovirus/Rhinovirus |
|  |  | Human Metapneumovirus |
|  |  | Influenza A/B |
|  |  | Parainfluenza 1-4 |
|  |  | Respiratory Syncytial Virus |
|  |  | SARS-CoV2 (COVID-19) |
|  |  | Seasonal Coronavirus |
|  |  | *Bordetella pertussis* and *parapertussis* |
|  |  | *Chlamydophila pneumoniae* |
|  |  | *Mycoplasma pneumoniae* |
| **Urinary Antigen Targets** | | |
| *Streptococcus pneumoniae* Antigen | 26.28% | *Streptococcus pneumoniae* |
| *Legionella pneumophila* Antigen | 29.40% | *Legionella pneumophila* serogroups 1-6 |

| **Supplemental Table 4.** Types of microbiologic tests included in the study. These fields are those present in the electronic health record | |
| --- | --- |
| **Frequency Blood Cultures AND/OR Respiratory Culture Obtained (84.6%)**  **N = 4,558** | |
| **Respiratory Cultures** | |
| Specimen Type | Body Site |
| Not Blood | Sputum |
| Body Fluid | Pleural |
|  | Pleural-Right |
|  | Pleural-Left |
| Bronchial | Bronchoalveolar Lavage (BAL)-Left Lower Lobe |
|  | BAL-Left Upper Lobe |
|  | BAL-Lingula |
|  | BAL-Right Lower Lobe |
|  | BAL-Right Middle Lobe |
|  | BAL-Right Upper Lobe |
|  | Bronchial Brush |
|  | Bronchial Lavage |
|  | Endotracheal Aspirate |
|  | Nasopharynx |
|  | Other |
|  | Sputum |
|  | Tracheal |
| Comment Specimen Submitted | Sputum |
|  | Sputum, Induced |
| Drainage | Pleural-Right |
| Endotracheal Aspirate | Endotracheal Aspirate |
|  | Sputum |
| Fine Needle Aspirate | Pleural-Left |
| Fluid | BAL-Left Lower Lobe |
|  | Endotracheal Aspirate |
|  | Pleural |
|  | Pleural-Left |
|  | Pleural-Right |
|  | Sputum |
|  | Sputum, Induced |
| Pleural Fluid-Right | Pleural-Right |
| Pleural Fluid | Pleural-Left |
|  | Thoracentesis |
| Respiratory | Sputum |
| Respiratory Secretions | Bronchial Wash |
|  | Bronchial Lavage |
|  | Endotracheal Aspirate |
|  | Lung-Left |
|  | Lung-Right |
|  | Mouth |
|  | Nose, External |
|  | Oral Cavity |
|  | Other |
|  | Sputum |
|  | Sputum, Induced |
|  | Tracheal |
|  | Tracheostomy Site |
|  | Transtracheal Aspirate |
| Sputum | Endotracheal Aspirate |
|  | Lung-Right |
|  | Mouth |
|  | Oropharynx |
|  | Sputum |
|  | Tracheal |
| Non-Swab | Sputum |
| Tissue | Pleural-Left |
| Tracheal Aspirate | Endotracheal Aspirate |
|  | Lung-Right |
|  | Tracheal |

| **Supplemental Table 5.** Univariate analysis for methicillin-resistant Staphylococcus aureus* | | | | |
| --- | --- | --- | --- | --- |
| **Risk Factor** | **All**  **N = 4,558** | **Non-MRSA**  **N = 4,531** | **MRSA**  **N = 27** | **p-value** |
| Bronchiectasis | 188 (4.1) | 188 (4.1) | 0 (0.0) | 0.627 |
| Chronic kidney disease | 1771 (38.9) | 1760 (38.8) | 11 (40.7) | 0.845 |
| End stage renal disease | 182 (4.0) | 178 (3.9) | 4 (14.8) | **0.021** |
| Influenza (Prior to admission) | 722 (15.8) | 715 (15.8) | 7 (25.9) | 0.180 |
| Concurrent influenza | 70 (1.5) | 70 (1.5) | 0 (0.0) | 1.000 |
| Diabetes (Type II) | 1740 (38.2) | 1728 (38.1) | 12 (44.4) | 0.553 |
| Chronic obstructive pulmonary disease | 1615 (35.4) | 1606 (35.4) | 9 (33.3) | 1.000 |
| Current or former tobacco/nicotine use | 3143 (69.6) | 3121 (69.5) | 22 (84.6) | 0.132 |
| Alcohol abuse | 750 (16.5) | 743 (16.4) | 7 (25.9) | 0.191 |
| Substance Use Disorders | 431 (9.5) | 424 (9.4) | 7 (25.9) | **0.011** |
| Psychoactive drugs | 146 (3.2) | 144 (3.2) | 2 (7.4) | 0.214 |
| Opioids | 207 (4.5) | 205 (4.5) | 2 (7.4) | 0.349 |
| Cocaine | 40 (0.9) | 39 (0.9) | 1 (3.7) | 0.212 |
| Non-psychoactive drugs | 177 (3.9) | 173 (3.8) | 4 (14.8) | **0.019** |
| Stimulants | 65 (1.4) | 63 (1.4) | 2 (7.4) | 0.056 |
| Abscess of Lung/Mediastinum | 30 (0.7) | 30 (0.7) | 0 (0.0) | 1.000 |
| Pyothorax | 38 (0.8) | 38 (0.8) | 0 (0.0) | 1.000 |
| Wound treatment | 2399 (52.6) | 2387 (52.7) | 12 (44.4) | 0.442 |
| Immunodeficiency or HIV | 395 (8.7) | 392 (8.7) | 3 (11.1) | 0.505 |
| Any COVID-19 history | 787 (17.3) | 782 (17.3) | 5 (18.5) | 0.800 |
| Recent COVID-19 diagnosis | 226 (5.0) | 226 (5.0) | 0 (0.0) | 0.642 |
| Concurrent COVID-19 diagnosis | 707 (15.5) | 705 (15.6) | 2 (7.4) | 0.419 |
| ICU admission within 48 hours | 1064 (23.3) | 1060 (23.4) | 4 (14.8) | 0.367 |
| **T-tests and Fisher’s exact test were used to test for an association with cases of MRSA* | | | | |

| **Supplemental Table 6.** Secondary outcomes for methicillin-resistant Staphylococcus aureus versus non-methicillin resistant Staphylococcus aureus community acquired pneumonia. Median (IQR) reported for days prescribed and LOS and n (%) reported for categorical variables. Wilcoxon rank-sum test and Fisher’s exact test were used to test for differences between MRSA cases and non-MRSA cases. | | | | |
| --- | --- | --- | --- | --- |
|  | **All**  **N = 4,558** | **Non-MRSA**  **N = 4,531** | **MRSA**  **N = 27** | **p-value** |
| Ordered antibiotic | 4405 (96.6) | 4379 (96.6) | 26 (96.3) | 0.603 |
| Days of antimicrobial therapy | 4.0 (4.0) | 4.0 (4.0) | 4.0 (6.8) | 0.132 |
| Length of stay | 5.0 (6.0) | 5.0 (6.0) | 9.0 (12.5) | **0.005** |
| 30-day readmission | 591 (13.0) | 588 (13.0) | 3 (11.1) | 1.000 |
| 60-day mortality | 893 (19.6) | 882 (19.5) | 11 (40.7) | **0.012** |

| **Supplemental Table 7.** Univariate tests for Pseudomonas *aeruginosa** | | | | |
| --- | --- | --- | --- | --- |
| **Risk Factor** | **All**  **N = 4,558** | **Non-PSA**  **N = 4,528** | **PSA**  **N = 30** | **p-value** |
| Bronchiectasis | 188 (4.1) | 187 (4.1) | 1 (3.3) | 1.000 |
| Chronic kidney disease | 1771 (38.9) | 1766 (39.0) | 5 (16.7) | **0.013** |
| End stage renal disease | 182 (4.0) | 182 (4.0) | 0 (0.0) | 0.632 |
| Influenza (Prior to admission) | 722 (15.8) | 715 (15.8) | 7 (23.3) | 0.310 |
| Concurrent influenza | 70 (1.5) | 70 (1.5) | 0 (0.0) | 1.000 |
| Diabetes (Type II) | 1740 (38.2) | 1734 (38.3) | 6 (20.0) | **0.040** |
| Chronic obstructive pulmonary disease | 1615 (35.4) | 1600 (35.3) | 15 (50.0) | 0.124 |
| Current or former tobacco/nicotine use | 3143 (69.6) | 3118 (69.5) | 25 (83.3) | 0.114 |
| Alcohol abuse | 750 (16.5) | 743 (16.4) | 7 (23.3) | 0.321 |
| Substance Use Disorders | 431 (9.5) | 428 (9.5) | 3 (10.0) | 0.759 |
| Psychoactive drugs | 146 (3.2) | 146 (3.2) | 0 (0.0) | 1.000 |
| Opioids | 207 (4.5) | 204 (4.5) | 3 (10.0) | 0.153 |
| Cocaine | 40 (0.9) | 40 (0.9) | 0 (0.0) | 1.000 |
| Non-psychoactive drugs | 177 (3.9) | 177 (3.9) | 0 (0.0) | 0.630 |
| Stimulants | 65 (1.4) | 65 (1.4) | 0 (0.0) | 1.000 |
| Abscess of Lung/Mediastinum | 30 (0.7) | 30 (0.7) | 0 (0.0) | 1.000 |
| Pyothorax | 38 (0.8) | 37 (0.8) | 1 (3.3) | 0.223 |
| Wound treatment | 2399 (52.6) | 2388 (52.7) | 11 (36.7) | 0.098 |
| Immunodeficiency or HIV | 395 (8.7) | 394 (8.7) | 1 (3.3) | 0.512 |
| Any COVID-19 history | 787 (17.3) | 783 (17.3) | 4 (13.3) | 0.808 |
| Recent COVID-19 | 226 (5.0) | 226 (5.0) | 0 (0.0) | 0.401 |
| Concurrent COVID-19 | 707 (15.5) | 705 (15.6) | 2 (6.7) | 0.306 |
| ICU admission within 48 hours | 1064 (23.3) | 1056 (23.3) | 8 (26.7) | 0.666 |
| ^*T-tests and Fisher’s exact test were used to test for an association with cases of^ *^Pseudomonas aeruginosa^* | | | | |

| **Supplemental Table 8.** Secondary outcomes for *Pseudomonas aeruginosa* versus non- *Pseudomonas aeruginosa* community acquired pneumonia. Median (IQR) reported for days prescribed and LOS and n (%) reported for categorical variables. Wilcoxon rank-sum test and Fisher’s exact test were used to test for differences between MRSA cases and non-MRSA cases. | | | | |
| --- | --- | --- | --- | --- |
|  | **All**  **N = 4,558** | **Non-PSA**  **N = 4,528** | **PSA**  **N = 30** | **p-value** |
| Ordered antibiotic | 4405 (96.6) | 4376 (96.6) | 29 (96.7) | 1.000 |
| Days of antimicrobial therapy | 4.0 (4.0) | 4.0 (4.0) | 4.0 (5.0) | 0.532 |
| Length of Stay | 5.0 (6.0) | 5.0 (6.0) | 5.5 (4.0) | 0.995 |
| 30-day readmission | 591 (13.0) | 583 (12.9) | 8 (26.7) | **0.048** |
| 60-day mortality | 893 (19.6) | 889 (19.6) | 4 (13.3) | 0.493 |

| **Supplemental table 9.** Post-hoc multivariate regression analysis for risk of methicillin-resistant *Staphylococcus aureus* | | | | | | |
| --- | --- | --- | --- | --- | --- | --- |
|  | All Patients (Planned Analysis) | | | Excluding Patients ≥ 72 Years | | |
| **Risk Factor** | **OR** | **95%CI** | **p-value** | **OR** | **95%CI** | **p-value** |
| Concurrent COVID-19 diagnosis | 0.46 | 0.10-2.01 | 0.300 | 0.76 | 0.16-3.51 | 0.725 |
| Wound treatment | 0.57 | 0.25-1.30 | 0.182 | **0.35** | **0.13-0.98** | **0.047** |
| ICU admission within 48 hours | 0.58 | 0.20-1.71 | 0.327 | 0.37 | 0.08-1.65 | 0.193 |
| Chronic obstructive pulmonary disease | 0.76 | 0.31-1.87 | 0.556 | 0.56 | 0.18-1.74 | 0.315 |
| Age (per 5 years) | **0.86** | **0.76-0.98** | **0.021** | 0.98 | 0.95-1.02 | 0.383 |
| Male | 1.07 | 0.48-2.40 | 0.862 | 1.29 | 0.49-3.41 | 0.602 |
| Any COVID-19 history | 1.16 | 0.42-3.22 | 0.773 | 0.53 | 0.11-2.49 | 0.424 |
| Immunodeficiency or HIV | 1.18 | 0.35-4.06 | 0.787 | 1.01 | 0.22-4.64 | 0.993 |
| Alcohol abuse | 1.23 | 0.47-3.21 | 0.670 | 1.13 | 0.37-3.41 | 0.828 |
| Diabetes (Type II) | 1.43 | 0.61-3.36 | 0.412 | 1.07 | 0.37-3.09 | 0.907 |
| Chronic kidney disease | 1.55 | 0.63-3.83 | 0.342 | 2.58 | 0.85-7.79 | 0.093 |
| Influenza (prior to admission) | 1.83 | 0.74-4.54 | 0.194 | 2.75 | 0.97-7.75 | 0.056 |
| Current or former tobacco/nicotine use | 2.21 | 0.72-6.80 | 0.165 | 3.78 | 0.82-17.49 | 0.089 |
| Substance use disorder^1^ | 2.38 | 0.90-6.25 | 0.080 | **3.01** | **1.05-8.68** | **0.041** |
| ^*Risk factors that were infrequent are not shown due to poor precision^  ^1Includes opioids (207), non-psychoactive drugs (177), psychoactive drugs (146), stimulants (65), and cocaine (40)^ | | | | | | |

**Supplemental Table 10. Viral-bacterial Coinfections**

|  | **COVID-19** | **Enterovirus-Rhinovirus** | **Influenza A** | **Seasonal Coronavirus** | **Parainfluenza Virus 1** | **Parainfluenza Virus 3** | **Parainfluenza Virus 4** | **Respiratory Syncytial Virus** | **Grand Total** |
| --- | --- | --- | --- | --- | --- | --- | --- | --- | --- |
| **Clostridium species** | 1 |  |  |  |  |  |  |  | **1** |
| **Cryptococcus species** |  |  |  |  |  |  |  | 1 | **1** |
| **Enterobacter cloacae** |  | 1 |  |  |  |  |  | 1 | **2** |
| **Escherichia coli** | 1 |  | 1 |  |  |  |  | 1 | **3** |
| **Fusobacterium species** | 1 |  |  |  |  |  |  |  | **1** |
| **Group A Streptococci** |  | 1 |  |  |  |  |  |  | **1** |
| **Group B Streptococci** |  | 1 |  |  |  |  |  |  | **1** |
| **Haemophilus influenzae** | 1 |  |  |  |  |  |  |  | **1** |
| **Klebsiella oxytoca** |  | 1 |  |  |  |  |  |  | **1** |
| **Klebsiella pneumoniae** | 1 |  |  |  |  |  |  |  | **1** |
| **Legionella pneumophila** |  | 3 |  |  |  |  |  |  | **3** |
| **MRSA** | 2 |  |  |  |  |  |  |  | **2** |
| **MSSA** | 4 | 1 |  |  |  |  |  |  | **5** |
| **Pseudomonas aeruginosa** | 1 |  |  |  |  |  | 1 |  | **2** |
| **Salmonella species** |  | 1 |  |  |  |  |  |  | **1** |
| **Streptococcus pneumoniae** | 3 | 3 | 2 | 2 | 1 | 1 |  | 2 | **14** |

**Supplemental Figure 1.** Forest plot for multivariate regression analysis of methicillin-resistant *Staphylococcus aureus*


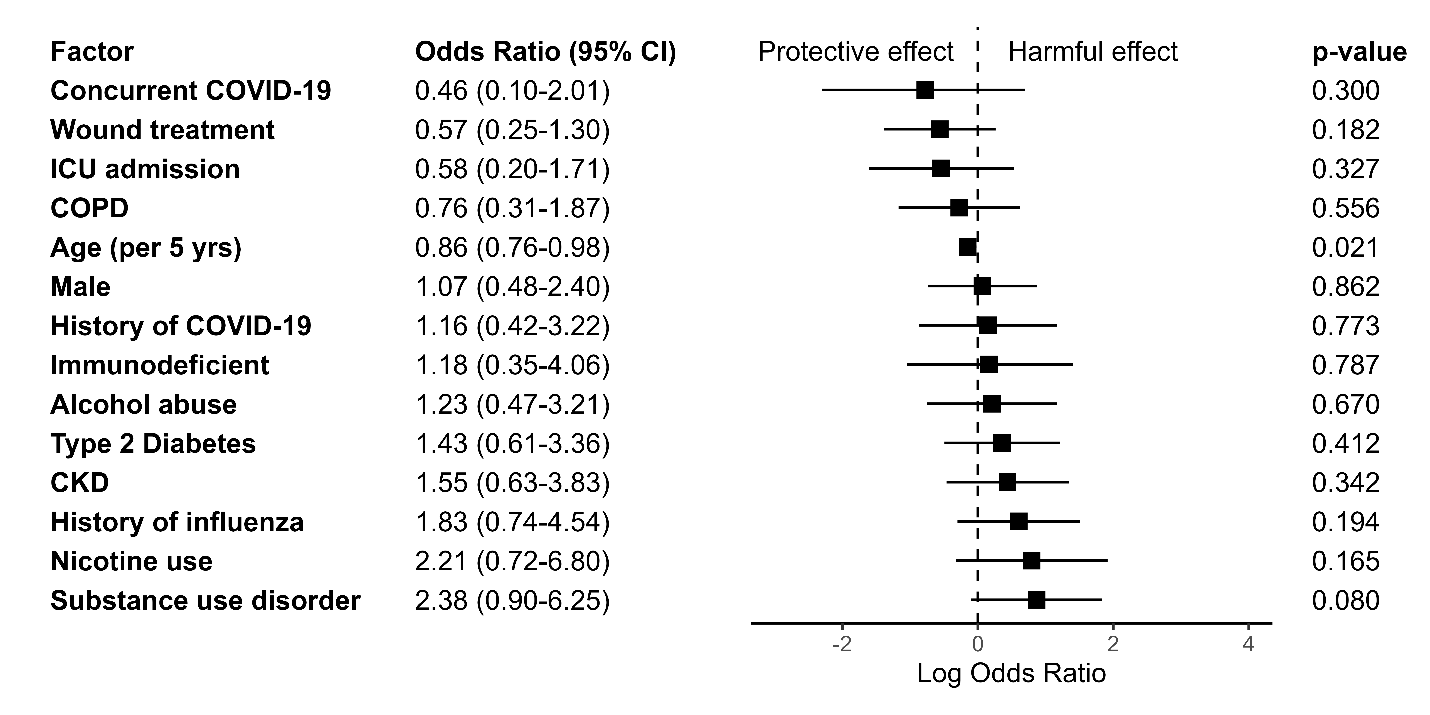


COPD; chronic obstructive pulmonary disease, CKD; chronic kidney disease, substance use disorder; includes opioids, non-psychoactive drugs, stimulants, and cocaine.

**Alt Text:** A graphical representation of Table 2 in the form of a forest plot showing the multivariate regression analysis performed for odds of MRSA pneumonia. Only age (per 5 years) was found to be inversely proportional to risk of MRSA pneumonia.

**Supplemental Figure 2.** Forest plot for multivariate regression analysis of *Pseudomonas aeruginosa*


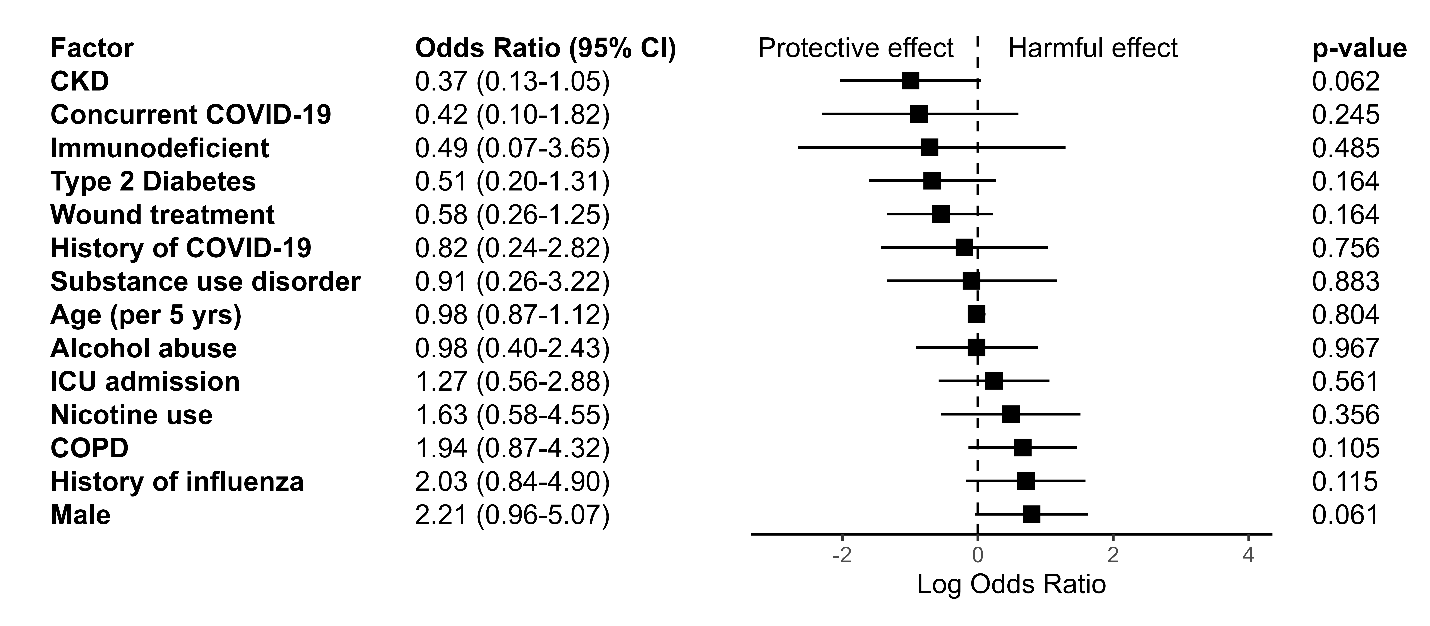


CKD; chronic kidney disease, substance use disorder; includes opioids, non-psychoactive drugs, stimulants, and cocaine, COPD; chronic obstructive pulmonary disease.

**Alt Text:** A graphical representation of Table 2 in the form of a forest plot showing the multivariate regression analysis performed for odds of *Pseudomonas aeruginosa* pneumonia. The forest plot shows no statistically significant positive or negative odds of *Pseudomonas aeruginosa* across all examined risk factors.
